# The interplay of maturity-onset diabetes of the young, obesity, uncontrolled hypertension, and cannabinoid hyperemesis in the progression to end-stage renal disease: a case report

**DOI:** 10.3389/fmed.2026.1755934

**Published:** 2026-05-01

**Authors:** Neguemadji Ngardig Ngaba, Victoria Lovallo, Anna ONeil, Xegfred Lou T. Quidet, Michael Asare, Henry Nabeta, Shagun Thakur, Riddick Osei Agyemang, Rayan Alataa, Fouad Kaddour Hocine, Karla Garcia Rodriguez, Abel Akanyijuka, Dennis Ansah, Akua Amoa, Oserefuamen Okobi, Shazia Khan, Ruchita Kodakandla, Sahil Dudhat, Shrushti Tanna, Del Orbe Eduardo, Muhammad Saleem Akhter, Rabih Nasr, Kalpana Uday, Khaja Misbahuddin

**Affiliations:** 1 Lake Chad Basin Commission, N'Djamena, Chad; 2 BronxCare Health System, Bronx, NY, United States

**Keywords:** cannabinoid hyperemesis, end stage renal disease, hypertension, MODY (mature onset diabetes of the young), obesity

## Abstract

**Background:**

Maturity-onset diabetes of the young (MODY) is a rare monogenic diabetes with significant renal risk, especially when combined with hyperglycemia, obesity, and other factors. While chronic cannabis use may further accelerate kidney decline, no previous reports have described rapid progression to ESRD in a young patient with MODY, obesity, hypertension, and cannabinoid hyperemesis, as presented here.

**Case presentation:**

A 24-year-old man with class II obesity, uncontrolled presumed MODY, CKD stage 5, diabetic gastroparesis, hypertension, and chronic marijuana use was diagnosed with MODY at age 18 after hospitalization for cannabinoid hyperemesis and Boerhaave syndrome. Despite insulin and metformin therapy, he experienced recurrent hypertensive crises and AKI with progressive renal decline. Over 70 months, the patient experienced progressive renal and metabolic deterioration. Serum creatinine increased from 0.9 to 10.8 mg/dL (mean 2.92), with eGFR declining from 115.98 to 4.69 mL/min/1.73 m^2^ (mean 52.75). Glycemic control was suboptimal (mean HbA1c 9.79%, range 6.5–12.1%). Urine microalbumin increased from 1.2 to 138, and the microalbumin/creatinine ratio increased from 32 to 931. BMI increased from 22.4 to 37.9 kg/m^2^, and toxicology screens were consistently positive for cannabinoids. Insulin aspart was initiated 1 month after presentation. By month 13, enalapril and scheduled prandial insulin were added; the secondary hypertension workup was negative despite suppressing aldosterone and low-normal renin. At month 30, enalapril was replaced with carvedilol and amlodipine, with increased renin but persistently suppressed aldosterone; imaging showed preserved EF, mild pulmonary hypertension, and grade 1 diastolic dysfunction. Hydralazine was added at month 34. By month 70, sitagliptin and sevelamer were initiated for diabetes and hyperphosphatemia (phosphorus 7.7 mg/dL), with persistently suppressed aldosterone (<1) and renin 4.97 ng/mL.

**Conclusion:**

This first reported case of presumed MODY, obesity, hypertension, cannabis use, and poor adherence in a young adult demonstrates unusually rapid progression to end-stage renal disease (ESRD), highlighting the need for early recognition and integrated management in high-risk patients.

## Introduction

Maturity-onset diabetes of the young (MODY) was first reported in 1974 by Tattersall and Fajans (1) with different inheritance patterns in the families of diagnosed patients.

MODY is a rare inherited form of diabetes caused by a single-gene mutation that affects insulin production (2, 3).

Meaningful studies about MODY in Germany reported an incidence of 2.4% and a prevalence of 23.9 per million (4, 5). In the US, the SEARCH for Diabetes in Youth Study found an estimated prevalence of 1.2% (6).

Hyperglycemia causes renal damage by activating protein kinase C, increasing advanced glycation end products, and stimulating diacylglycerol synthesis, while also inducing hemodynamic changes, such as glomerular hyperfiltration, shear stress, and microalbuminuria (7).

Obesity-related kidney disease is driven by several mechanisms, broadly categorized into three main pathways: hemodynamic alterations, adipose tissue-related effects, and insulin resistance (8–10).

It has been shown that cannabinoid (CB) receptors are present in peripheral organs such as the kidneys (11). The impact of cannabis on kidney health remains highly controversial. While cannabis may provide therapeutic benefits for patients with kidney impairment (12), recent evidence—including findings from the ASSESS-AKI study (13)—indicates that chronic cannabis use in individuals with reduced eGFR (<60 mL/min/1.73 m^2^) may be linked to a more rapid decline in kidney function compared to those with normal renal function.

To our knowledge, there are no reported cases of a young patient with the combination of MODY, obesity, and cannabinoid use resulting in cannabinoid hyperemesis syndrome, recurrent acute kidney injury (AKI) on multiple admissions, and progression to ESRD within 5 years of MODY diagnosis.

We report a case of a 24-year-old man with class II obesity, CKD stage 5, uncontrolled presumed MODY, diabetic gastroparesis, hypertension, and chronic marijuana use. He was presumptively diagnosed with MODY at 19 years of age, with multiple admissions for hypertensive crises and cannabinoid hyperemesis-induced AKI. Over 70 months, his serum creatinine increased from 0.9 to 10.8 mg/dL, eGFR dropped from 115.98 to 4.69 mL/min/1.73 m^2^, and HbA1c remained poorly controlled. Urine microalbumin and microalbumin/creatinine ratios increased significantly, and BMI increased from 22.4 to 37.9 kg/m^2^. Persistent cannabinoid use and episodes of hyperemesis contributed to volume depletion and prerenal azotemia, accelerating progression from CKD to ESRD in the setting of poor glycemic control and increasing obesity.

### Case description

A 24-year-old man, obese class II (109.8 kg, BMI 37.9) with a past medical history of pyelonephritis, chronic kidney disease stage 5 (CKD 5), poorly controlled presumed MODY, diabetic gastroparesis, hypertension (HTN), upper GI bleed due to Mallory–Weiss tear, chronic marijuana use, cyclic vomiting syndrome, and mild hiatal hernia presented to the emergency department for diffuse abdominal pain (6/10), nausea, vomiting, epigastric pain, and anorexia. At the time of presentation to the emergency department, the patient was tachycardic (HR 121 bpm) and hypertensive (BP 168/114 mmHg). Laboratories were significant for mild leukocytosis, metabolic alkalosis, severe acute kidney injury with creatinine above baseline, and hyperglycemia.

The patient was born with macrosomia (14), weighing 11 pounds, and was delivered via a lower-segment cesarean section without complications.

**Figure 1:**
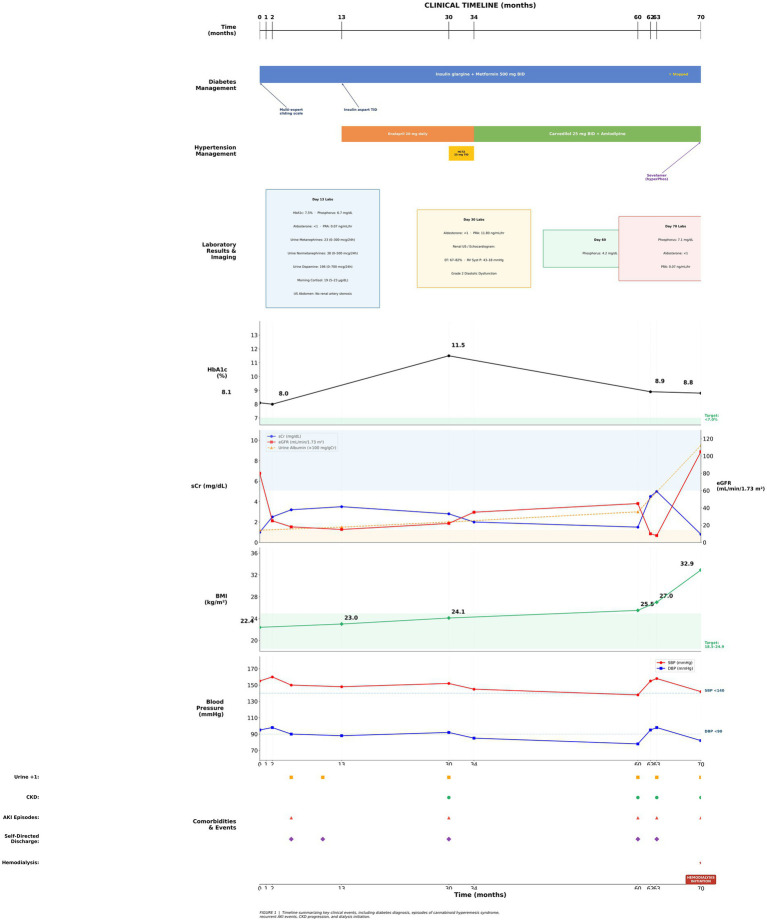
Timeline summarizing key clinical events, including diabetes diagnosis, episodes of cannabinoid hyperemesis syndrome, recurrent AKI events, CKD progression, and dialysis initiation.

At 19 years of age, he was hospitalized with newly diagnosed diabetes mellitus (DM) and subsequently presumed to have MODY after etiologic evaluation, following an emergency department visit for hyperemesis cannabinoid syndrome, attributed to cannabis abuse, and pneumomediastinum resulting from Boerhaave syndrome. Laboratory findings (Figure 1) at that time indicated significant hyperglycemia (511 mg/dL) and an elevated glycated hemoglobin A1C (HbA1c) of 11.5% (reference range 4.7–6.4%). Additional laboratory results showed a normal glutamic acid decarboxylase that was <5 (normal value <5 IU/mL) with an elevated C-peptide level of 3.9 (reference range 0.8–31 ng/mL), serum creatinine (sCr) of 1.9 mg/dL, an estimated glomerular filtration rate (eGFR) of 36.68 mL/min, blood urea nitrogen (BUN) of 36 mg/dL, and microalbuminuria at 32 mg/g. The patient’s BMI was 22.4 kg/m^2^ at this time (height 172 cm and weight 65.4 kg). Three years later, the patient was admitted for prerenal acute kidney injury in the context of dehydration due to cannabinoid hyperemesis syndrome, accompanied by hypertensive urgency.

The patient was repeatedly educated during hospital visits on identifying hypoglycemia symptoms and the importance of medication adherence for diabetes and hypertension to prevent complications such as diabetic nephropathy and AKI. Counseling was also provided on the benefits of quitting marijuana to reduce dependency and recurrent symptoms. The patient is known to have a tendency to self-direct discharge from the hospital (Figure 1).

Over several admissions from ages 19–24 (Figure 1), various parameters were monitored, including kidney function, HbA1c, toxicology screenings, and anthropometric measurements from baseline to month 70. The average sCr was 2.92 mg/dL (normal range 0.6–1.3 mg/dL) with the lowest value of 0.9 mg/dL in the first month and the highest value of 10.8 mg/dL in month 70. The mean estimated glomerular filtration rate (eGFR) was 52.75 mL/min/1.73 m^2^ (normal is ≥90 mL/min/1.73 m^2^), with the highest at 115.98 mL/min/1.73 m^2^ on the first day of the hospital visit and the lowest at 4.69 mL/min/1.73 m^2^ in month 70. The average HbA1c was 9.79%, peaking at 12.1% and reaching a low of 6.5% when kidney function declined, as indicated by a GFR of 4.69 mL/min/1.73 m^2^ in month 70. Urine microalbumin levels increased from 1.2 mg/dL (normal is <1.2 mg/dL) at baseline to 138 mg/dL by month 63 following the diagnosis of MODY. The microalbumin/creatinine ratio rose from 32 mg/gm (normal 30–299 mg/gm) at baseline to 931 mg/gm by month 63. Urine tests for cannabinoids were consistently positive during hospital visits.

One month after the initial presentation, the insulin aspart sliding scale was initiated, with a phosphorus level of 2.9 mg/dL. By month 13, enalapril 20 mg daily and scheduled prandial insulin aspart were added; phosphorus remained at 2.9 mg/dL. Evaluation for secondary HTN was unrevealing, including normal renal artery Doppler, suppressed aldosterone (<1), low-normal plasma renin activity (0.67 ng/mL), normal urinary metanephrines/normetanephrines, and normal morning cortisol (19 μg/dL).

At month 30, enalapril was discontinued and replaced with carvedilol and amlodipine; aldosterone remained suppressed, while plasma renin activity increased to 11.86 ng/mL. Renal ultrasound was normal, and the echocardiogram showed preserved EF (67.82%), mild pulmonary pressure elevation (RVSP 43.18 mmHg), and grade 1 diastolic dysfunction. Hydralazine was added at month 34 to further lower blood pressure.

By month 70, sitagliptin was added for diabetes management, and sevelamer was initiated for hyperphosphatemia, with phosphorus rising to 7.7 mg/dL. Aldosterone remained <1, and plasma renin activity was 4.97 ng/mL.

Five years after the initial presumptive diagnosis of MODY and 3 years following the diagnosis of CKD, the patient was admitted for cannabinoid hyperemesis syndrome and severe CKD stage 5 (serum creatinine 9.31 mg/dL, urine protein-to-creatinine ratio 1.4 g/g). During this admission, a permacath was placed, and the patient was initiated on hemodialysis as per the guidelines (15, 16). He is scheduled for hemodialysis three times per week (Tuesday, Thursday, and Saturday) via the permcath. He was discharged with follow-up appointments arranged: primary care within 1 week, nephrology within 1 month, and gastroenterology within 2 months.

## Discussion

The disease history of our patient emphasizes many key challenges in the diagnosis and management of rare diabetes subtypes and their multisystem complications.

The birth history of the patient revealed a finding of macrosomia with a birth weight of 11 pounds. This finding is consistent with a study by Pearson et al. (17) in the United Kingdom, which found a relationship between macrosomia at birth and MODY in their series.

The patient was initially presumed to have MODY at 19 years of age. The literature (18) found that MODY can be diagnosed at various ages, ranging from 11 to 55 years. The patient’s age of 19 falls within the age bracket reported in previous studies, such as those cited above.

The patient’s early-onset diabetes, absence of autoimmunity, preserved C-peptide, and family history are consistent with a MODY phenotype, though the specific genetic subtype was not determined in our case. MODY is frequently misclassified as type 1 or type 2 diabetes (19), leading to suboptimal management and delayed recognition of associated comorbidities. This case illustrates the need for increased alertness and the use of clinical biomarkers (such as persistent C-peptide and negative autoantibodies) to guide the establishment of an appropriate diagnosis and the further management of MODY.

There is a notable rapid progression from microalbuminuria to ESRD requiring hemodialysis within 5 years for our patients with MODY.

Possible hypertensive nephrosclerosis caused by chronic hypertension and diabetes (which includes MODY) synergistically led to kidney damage and rapid progression to ESRD in our patient, as discussed in meaningful literature by Marks and Raskin (20). According to large-scale studies (21, 22), in patients with diabetes (which includes MODY), it takes approximately 10–20 years for the progression to ESRD from microalbuminuria, but it can be earlier in some cases, depending on glycemic control and other risk factors (HTN, lifestyle, and adherence). The severity and speed of decline in this patient may reflect the combined impact of poor glycemic control (as evidenced by persistently elevated HbA1c), recurrent episodes of dehydration from cannabinoid hyperemesis inducing AKI, and possible hypertensive nephrosclerosis.

Chronic marijuana use and recurrent self-directed discharge from the hospital significantly complicate disease management. Cannabinoid hyperemesis syndrome contributed to repeated episodes of dehydration and acute kidney injury, as reported in the literature (23) and a previous case report (24), accelerating CKD progression. Regarding our patient, the development of CKD following recurrent AKI episodes is consistent with findings by Charlton et al. (25), who demonstrated progression from AKI to CKD by longitudinally tracking renal pathology using MRI. The patient’s non-adherence to medication and follow-up, despite repeated counseling, reflects the psychosocial burden and behavioral challenges common in young adults with chronic disease. Studies^1^ (26) show that youth with atypical diabetes are subject to higher rates of psychiatric comorbidity and diabetes-specific distress, which can further impair self-management and outcomes. Integrating mental health support and substance use counseling into diabetes care is essential for improving adherence and long-term prognosis.

The patient’s transition from a normal BMI at diagnosis to class II obesity over 5 years. This finding is consistent with a study (27) that reported obesity and obesity-induced insulin resistance in subjects with MODY. The obesity in our patient worsened insulin resistance, marked by consistently elevated HbA1c.

### Limitations

The absence of genetic testing precluded definitive confirmation and subtype classification of MODY; however, the diagnosis was strongly supported by early disease onset and the exclusion of more common forms of diabetes. Progressive renal dysfunction developed in the context of recurrent prerenal AKI, uncontrolled diabetes, and HTN, and more invasive evaluation (renal biopsy, autoimmune studies, or genetic testing for hereditary nephropathies) was not undertaken. As a result, renal outcomes could not be attributed to a specific MODY subtype, though the clinical trajectory and laboratory trends highlight a multifactorial progression from AKI to CKD. As a single-patient case report, this study is not intended to be generalizable but rather to underscore a clinically important and underrecognized presentation that warrants heightened awareness and early diagnostic consideration.

## Conclusion

This case highlights the complex and multifactorial progression of renal disease in a young patient with presumed MODY, obesity, chronic HTN, and persistent cannabinoid use. Despite early-onset diabetes with preserved C-peptide and negative autoantibodies suggestive of MODY, the absence of genetic confirmation limited precise subtype classification and risk stratification.

Over 5 years, he experienced rapid deterioration from microalbuminuria to ESRD, a trajectory likely driven by poorly controlled hyperglycemia, recurrent cannabinoid hyperemesis-associated AKI, progressive obesity-related insulin resistance, and uncontrolled HTN.

This case underscores the importance of early recognition of MODY, aggressive management of modifiable risk factors (glycemic control, blood pressure, weight, substance use), and the integration of psychosocial support to prevent devastating complications.

To our knowledge, this represents the first reported case documenting the convergence of MODY, obesity, HTN, chronic cannabinoid use with hyperemesis syndrome, and rapid progression to ESRD in a young adult, highlighting a clinically important and underrecognized presentation that warrants heightened awareness in clinical practice.

## Data Availability

The raw data supporting the conclusions of this article will be made available by the authors, without undue reservation.

## Footnotes

1
https://www.cdc.gov/diabetes/data-research/research/young-people-mental-health.html#90a7d815
